# Maternal and Paternal Risk Factors for Hypospadias

**DOI:** 10.1289/ehp.113-1257578

**Published:** 2005-05

**Authors:** Sebastiano Bianca, Carmela Ingegnosi, Giuseppe Ettore

**Affiliations:** Dipartimento Materno Infantile, Centro di Consulenza Genetica e di, Teratologia della Riproduzione, ARNAS, P.O. Garibaldi Nesima, Catania, Italy, E-mail: sebastiano.bianca@tiscali.it; Dipartimento Materno Infantile, U.O. Ginecologia e Ostetricia, ARNAS, P.O. Garibaldi Nesima, Catania, Italy

Hypospadias is a common congenital anomaly, characterized by incomplete fusion of the urethral folds during fetal development, which results in the urethra opening on the ventral surface of the penis or on the scrotum. In their article, Pierik et al. (2004) proposed multiple possible maternal and paternal risk factors related to the development of isolated hypospadias, including genetic, endocrine, and environmental factors.

Concerns and alarms have been raised about the potential effects of endocrine disruptors, which include derivatives of poly-aromatic hydrocarbons and pesticides, on the developing male reproductive tract. We have recently published two case–control studies on risk factors and hypospadias, one on a possible association with maternal age (Bianca et al. 2005) and the other on the role of endocrine disruptors (Bianca et al. 2003).

In the first study (Bianca et al. 2005), we evaluated 415 newborns with isolated hypospadias and 812 controls. Our results suggest that an increased risk for hypospadias exists in women at the extremes of the age distribution (< 20 years and > 40 years; *p* = 0.000 and 0.026, respectively) relative to women in the middle of the distribution, with a mechanism probably related to hormonal disruption. It has been postulated that changes in concentrations of sex hormones during the fetal critical period of genital development (weeks 8–14), caused by endogenous or exogenous factors, may play a role in the development of hypospadias and that hypospadias could be associated with early malfunction of the placenta, resulting in decreased secretion of placental and fetal hormones that could in turn disturb fetal development (Akre et al. 1999). Mothers at the extremes of the age distribution may be more susceptible to this hormonal disruption. The association between hypospadias and maternal age, both for younger and older women, might be explained as a “defect in nature’s quality control” caused by a reduction of defensive maternal mechanisms that would be less efficient in the elimination of malformed fetuses in the mothers of hypospadias cases. This hypothesis may also apply to other birth defects where a relationship with maternal age has been demonstrated.

In the second study of 68 cases and 211 controls (Bianca et al. 2003), we identified a high incidence of hypospadias in two towns in southeastern Sicily, which have intense industrial (Augusta) and agricultural (Vittoria) activities. Our results showed an incidence that was 3.8 [95% confidence interval (CI), 2.16–6.14] and 2.3 times (95% CI, 1.48–3.43) higher, respectively, than expected (3.2 per 1,000 male live births in southeastern Sicily). The odds ratios for fathers’ job exposure alone were 5.5 (95% CI, 1.22–24.7) for working in an oil refinery in Augusta and 2.9 (95% CI, 1.01–8.55) for working in hothouses in Vittoria.

Pollutants in both areas include compounds with proven estrogenic activity and other chemicals: pesticides and herbicides and their metabolites; dieldrin, chlordane, and endosulfan; polychlorinated biphenyls and dioxins; bisphenols used in epoxy resins; and some phthalates used as plasticizers in a wide variety of applications, including polyvinyl compounds and alkyphenol ethoxylates. The direct cause–effect relationship between environmental pollutants, which act as endocrine disruptors, and the increased incidence of hypospadias in these areas is difficult to establish and demonstrate.

Our study (Bianca et al. 2003), suggested that exposure to large amounts of industrial and agricultural pollutants is sufficient to increase the risk of hypospadias. Other risk factors, such as reproductive history, may be involved in the etiology of hypospadias. Disturbances in sexual differentiation occur when endogenous and/or exogenous factors act to disrupt the metabolism of gonadal hormones during development.

Further epidemiologic and biologic studies are needed to explain, in a multi-factorial model, which factors (genetic and/or environmental) interact and influence the etiology of this congenital abnormality.
